# Application of DCE-MRI radiomics signature analysis in differentiating molecular subtypes of luminal and non-luminal breast cancer

**DOI:** 10.3389/fmed.2023.1140514

**Published:** 2023-04-25

**Authors:** Ting Huang, Bing Fan, Yingying Qiu, Rui Zhang, Xiaolian Wang, Chaoxiong Wang, Huashan Lin, Ting Yan, Wentao Dong

**Affiliations:** ^1^Department of Radiology, Jiangxi Provincial People’s Hospital, The First Affiliated Hospital of Nanchang Medical College, Nanchang, China; ^2^Department of Pharmaceutical Diagnosis, GE Healthcare, Changsha, China

**Keywords:** radiomics signature, molecular subtypes, luminal, breast cancer, magnetic resonance imaging

## Abstract

**Background:**

The goal of this study was to develop and validate a radiomics signature based on dynamic contrast-enhanced magnetic resonance imaging (DCE-MRI) preoperatively differentiating luminal and non-luminal molecular subtypes in patients with invasive breast cancer.

**Methods:**

One hundred and thirty-five invasive breast cancer patients with luminal (*n* = 78) and non-luminal (*n* = 57) molecular subtypes were divided into training set (*n* = 95) and testing set (*n* = 40) in a 7:3 ratio. Demographics and MRI radiological features were used to construct clinical risk factors. Radiomics signature was constructed by extracting radiomics features from the second phase of DCE-MRI images and radiomics score (rad-score) was calculated. Finally, the prediction performance was evaluated in terms of calibration, discrimination, and clinical usefulness.

**Results:**

Multivariate logistic regression analysis showed that no clinical risk factors were independent predictors of luminal and non-luminal molecular subtypes in invasive breast cancer patients. Meanwhile, the radiomics signature showed good discrimination in the training set (AUC, 0.86; 95% CI, 0.78–0.93) and the testing set (AUC, 0.80; 95% CI, 0.65–0.95).

**Conclusion:**

The DCE-MRI radiomics signature is a promising tool to discrimination luminal and non-luminal molecular subtypes in invasive breast cancer patients preoperatively and noninvasively.

## 1. Introduction

Greater numbers of younger patients are suffering from breast cancer, and its mortality rate ranks first among female malignant tumors in recent years (1, 2). The molecular subtypes of breast cancer play an important role in clinical treatment decisions. According to four indicators of the expression levels of certain receptors (ER, PR, Her-2, and Ki-67), the molecular subtypes of breast cancer are classified into luminal A, luminal B, human epidermal growth factor receptor 2 (HER2) -enriched and triple-negative (3–5). Generally, luminal types account for the majority (about 70%) of invasive breast cancers and respond well to endocrine therapy (6, 7). The selection of targeted antibody therapy is a priority for HER2-enriched types (8). Triple-negative breast cancer, which is negative for hormone receptor and HER-2, loses the opportunity to use endocrine drugs and targeted therapy, and can only be further treated by chemotherapy and radiotherapy, and has the worst prognosis (9, 10). In conclusion, luminal types in invasive breast cancer have the best prognosis, and its hormone receptor positivity gives us the opportunity to treat with endocrine drugs that do the least harm to the body. Therefore, there is an urgent need for a noninvasive and efficient method to differentiate luminal and non-luminal molecular subtypes in invasive breast cancer patients before surgery.

For non-invasive diagnosis, various imaging techniques can be used, including mammography, ultrasound, MRI, etc. DCE-MRI is a satisfactory imaging modality, which can provide temporal information of the contrast agent dynamics of suspected lesions and acceptable spatial resolution. It is a sensitive sequence for the detection of breast cancer lesions, especially for dense breast lesions (11, 12). Tumor angiogenesis is a marker of tumor invasion potential. DCE-MRI can indirectly reflect tumor abnormal vascular proliferation through the hemodynamic characteristics of the lesion (13, 14). However, DCE-MRI has limited predictive power for luminal and non-luminal molecular subtypes in patients with invasive breast cancer, and diagnostic accuracy based on DCE-MRI images is largely dependent on the radiologist’s experience, which is highly subjective (15–17).

The development of artificial intelligence has made it possible to integrate the experience of imaging diagnosis, and various advanced medical image analysis methods have come into being. The research in related fields has improved the accuracy of disease diagnosis and can assist radiologists to carry out their daily work accurately and efficiently (18–20). The semi-quantitative or quantitative information of morphology provided by routine imaging examination could not fully meet the needs of precision medicine (21). In recent years, the emergence of radiomics provides a new method for evaluating tumor characteristics. Radiomics is one of the applications of artificial intelligence in medical field, it uses medical image analysis tools to extract some features of images (such as gray level) and create a digital matrix to find the association between voxels in images (22–24).

The purpose of this study was to evaluate the ability of DCE-MRI radiomics signature to noninvasively distinguish luminal and non-luminal molecular subtypes of invasive breast cancer patients.

## 2. Materials and methods

### 2.1. Patients

**Figure 1:**
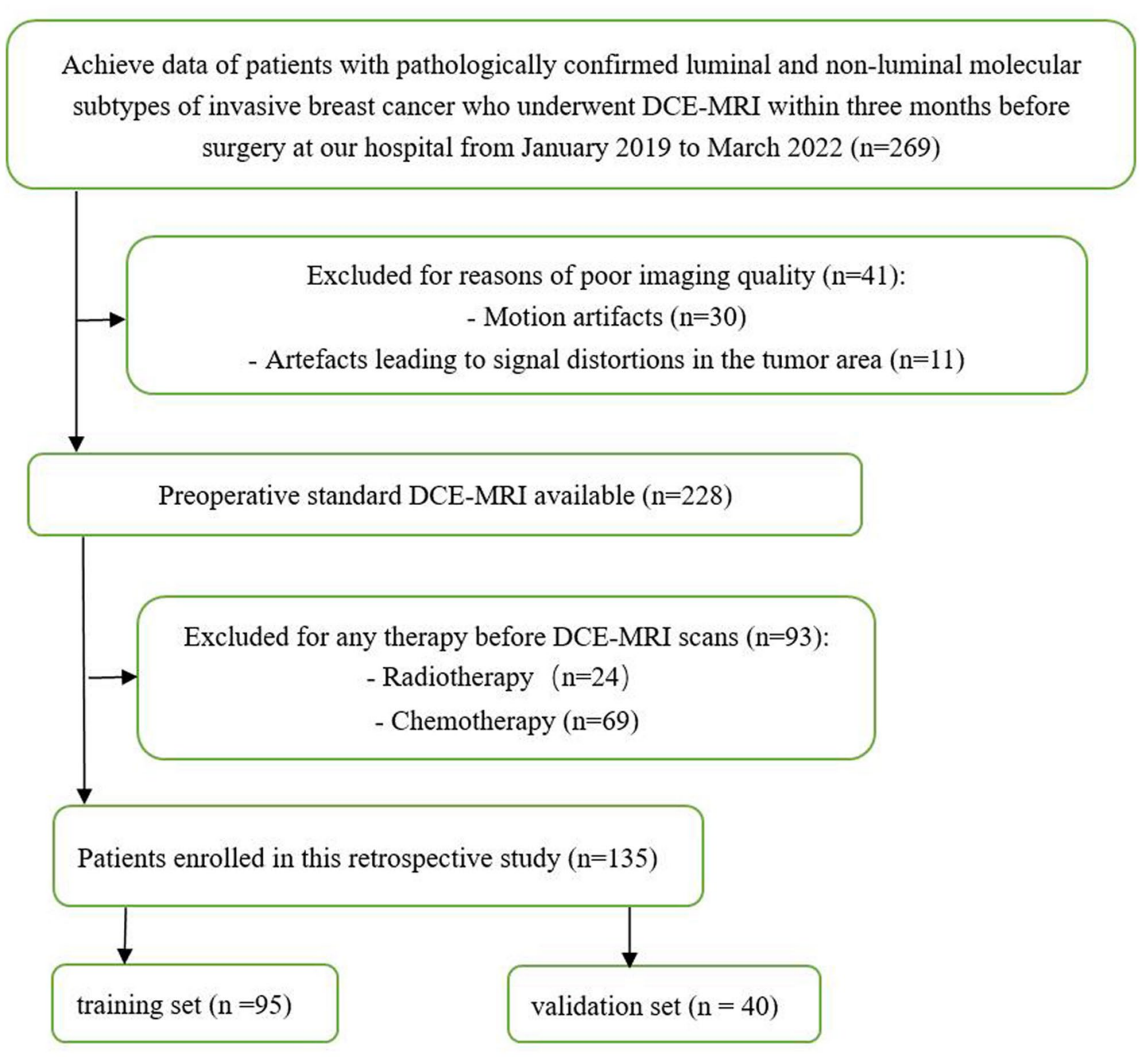
Flow diagram of the study.

This retrospective study included patients with luminal and non-luminal molecular subtypes of invasive breast cancer who underwent DCE-MRI at our hospital from January 2019 to March 2022. The inclusion criteria were as follows: (1) primary invasive breast cancer that underwent surgery, such as biopsy or resection, (2) histological diagnosis and molecular subtypes of breast cancer were obtained, and (3) DCE-MRI was performed within 3 months before surgery. The exclusion criteria were as follows: (1) patients received radiotherapy or chemotherapy before DCE-MRI scans and (2) poor imaging quality making difficulties in segmentation (e.g., motion artifacts or artefacts leading to signal distortions in the tumor area). Finally, a total of 135 luminal (78 cases, ages 33–79 years) and non-luminal (57 cases, ages 28–70 years) molecular subtypes of invasive breast cancer were included in this study (Figure 1; Table 1).

**Table 1:** Histologically confirmed tumor distribution in the whole cohort.

Luminal	Number	Non-luminal	Number
Luminal A	47	(HER2) -enriched	31
Luminal B	31	triple-negative	26

Patients were randomly assigned to the training and testing sets in a ratio of approximately 7:3. The training and testing sets were stratified to maintain the same proportion of luminal and non-luminal molecular subtypes of tumors in the training and testing sets.

### 2.2. MRI image acquisition

MR Scans were performed using a SIMENS MAGNETOM SKYRA 3.0T-MR Scanner with an 18-channel dual-emulsion phased front coil. The patient was placed in a prone position, feet advanced, with bilateral breasts naturally hanging in the breast coil.

Scanning parameters: 1. axial T1WI (TR =735 ms, TE = 8.1 ms, slice thickness = 4 mm, slice spacing =1 mm), acquisition matrix 224 × 320, field of view 320 × 320 mm; 2. axial fat suppression T2WI (TR = 3,700 ms, TE = 101 ms, slice thickness = 4 mm, slice spacing = 1 mm), acquisition matrix 224 × 320, field of view 320 × 320 mm; 3. DCE scanning was performed on T1 fat suppression: TR 5.24 ms, TE 2.46 ms, layer thickness 1.5 mm, acquisition matrix 182 × 320, field of view 320 mm, turning Angle 10°. Before contrast injection, the mask was scanned, and then Gd-DTPA was injected into the dorsal vein through a high-pressure syringe with a dose of 0.1 mmol/kg and an injection flow rate of 2.5 mL/s. After contrast injection, 20 mL normal saline was rapidly injected. Then seven consecutive intervals were scanned.

### 2.3. MRI characteristic evaluation

The MRI image were scrutinized by two radiologists with 10 years (read 1) and 15 years (read 2) of diagnostic breast imaging experience. Blinded to the clinic-pathologic data, the two doctors interpreted the following MRI features by consensus: “Breast parenchymal pattern” was evaluated on the T1WI sequence using a semi-quantitative method according to ACR-BI-RADS-MRI (2013). “Maximum diameter” was the longest diameter of the tumor on an axial MRI image. “DCE-TIC”: The time-intensity curve (TIC) type of each case was plotted based on DCE-MRI, and an area of interest (ROI) of about 0.2–0.4 cm^2^ was placed on each piece of the brightest part of the lesion in the early images obtained after the injection of contrast agent. When each lesion had a different type, we recorded the type of high TIC curve. “MRI-determined presence of ALN metastasis”: All axillary lymph nodes were evaluated on T1 + C axial and coronal images. The morphologic criteria for evaluating ALN metastasis are as follows: absence of hilum structure, lymphatic hilum displacement, eccentric cortical thickening, short diameter >1 cm, or long diameter to short diameter ratio less than 2 (25, 26).

### 2.4. Assessed clinical risk factors

The clinical parameter of age was retrieved from the electronic medical record system of our hospital. Two radiologists with 10 and 15 years of experience in breast imaging were blinded to imaging reports and pathological details, and imaging features including breast parenchymal pattern, maximum diameter, DCE-TIC, and MRI-determined presence of ALN metastasis were reviewed and reported. When assessed clinical risk factors, the above demographics and MRI radiological features were analyzed by univariate regression, and then the statistically significant characteristics in the univariate regression analysis were processed by multivariate regression model. Ultimately, features with *p* < 0.05 were considered as independent predictors of luminal and non-luminal molecular subtypes in invasive breast cancer patients.

### 2.5. Tumor segmentation

**Figure 2:**
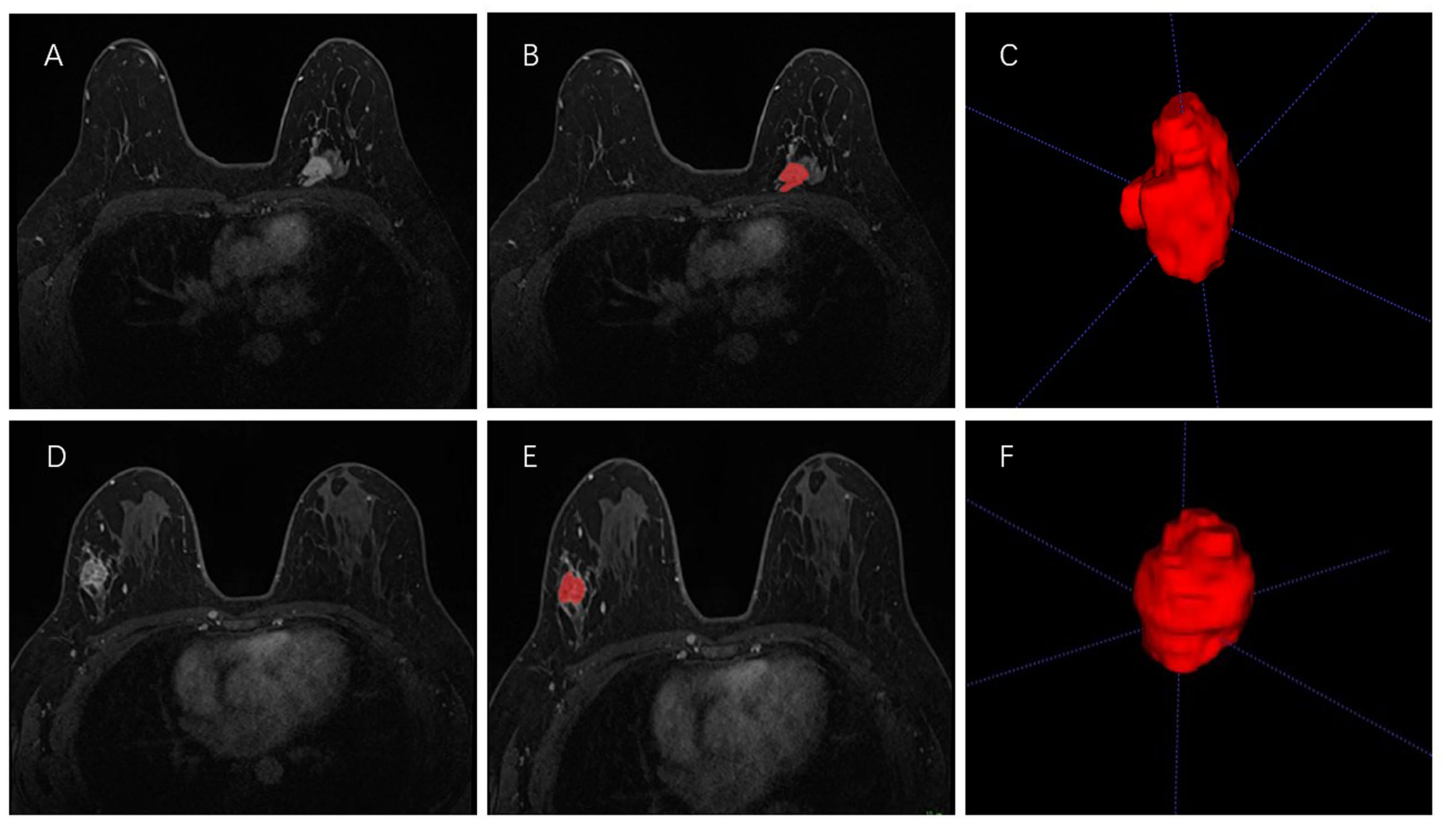
Manual 3-D segmentation of the tumor. **(A–C)**: a 58-year-old female diagnosed with the luminal A subtype of breast cancer. **(D–F)**: a 30-year-old female diagnosed with the triple-negative subtype of breast cancer. No special radiological features are seen by naked eye on MRI to distinguish between these two molecular subtypes.

All the second phase of DCE-MRI images in DICOM format, original size and resolution were transferred to ITK-SNAP software (Version 3.8, www.itksnap.org) for three-dimensional (3D) region of interest (ROI) segmentation. To ensure accurate tumor boundaries, ROIs on all slices were carefully delineated manually by a radiologist (read 1) with 10 years of experience in breast imaging, who was blinded to the pathological findings. To test the stability of features, doctor 1 and another radiologist with 15 years of experience (read 2) underwent re-extraction of radiomics features from 40 randomly selected patients from the entire study set. Intra-class correlation coefficient (ICC) was calculated to evaluate the consistency and reproducibility of the features. Subsequent analyses included features of ICC > 0.75 in intra-observer and inter-observer consistency analyses (27, 28). In order to avoid local volume effect, the top layer and bottom layer were eliminated. Figure 2 shows two typical the second phase of DCE-MRI images of breast cancer segmentation on ITK-SNAP, one of the molecular subtypes is luminal (Figures 2A–C) and the other is non-luminal (Figures 2D–F).

### 2.6. Radiomics feature extraction

Prior to radiomics feature extraction, to reduce feature variability, we performed the following image preprocessing steps, containing gray discretization, intensity normalization, and voxel resampling (29). Then, radiomics features were extracted from the second phase of DCE-MRI images through the open source PyRadiomics library. They are divided into four categories: size and morphology features, descriptors of image intensity histograms, descriptors of the relationship between image voxels and higher-order texture features extracted from filtered images.

### 2.7. Construction and assessment of the radiomics signature

In order to prevent signature overfitting, dimensionality of features is reduced before signature construction. Succinctly, radiomics features that met the inter-observer and intra-observer ICCs criteria greater than 0.75 and were significantly different between the two groups as assessed by one-way analysis of variance (ANOVA) were included in the LASSO regression model to select the most valuable features in the training set. Finally, the selected radiomics features were used to construct radiomics signature. Rad-score was calculated for each patient by linear combination of selected features and weighted by the respective LASSO coefficients, which was calculated using the following formula:

“Radscore = 0.423168579279913*(Intercept) + 0.256145048374 423*log_sigma_3_0_mm_3D_gldm_SmallDependenceHighGrayLevelEmphasis+0.189746981186449*lbp_3D_k_firstorder_Minimum+0.160418301883463*wavelet_HHL_glcm_Id+0.152274099347284*wavelet_LHH_glcm_MaximumProbability+0.046015260261054*lbp_3D_m1_glszm_SmallAreaLowGrayLevelEmphasis+ − 0.057685179007322*wavelet_HLL_glcm_Imc1 + −0.0877353138903384*wavelet_HLH_glszm_SmallAreaEmphasis+ − 0.0953377462671598*wavelet_LHH_glszm_SizeZoneNonUniformityNormalized+ −0.273401999050143*wavelet_HHH_glszm_SmallAreaLowGrayLevelEmphasis+ − 0.318474415701059*lbp_3D_m1_firstorder_InterquartileRange+ − 0.334402481515917*wavelet_LLH_glszm_SmallAreaEmphasis+ − 0.360310670738742*wavelet_HHL_glcm_Imc1 + −0.364519784506579*wavelet_LHH_firstorder_Skewness+ − 0.418315908802964*lbp_3D_k_glcm_MaximumProbability.”

Calibration curve was used to evaluate the calibration effect of radiomics signature. The Hosmer-Lemeshow test was used to evaluate the goodness of fit of radiomics signature. ROC curves of training and testing sets used to evaluate the diagnostic performance of radiomics signature in distinguishing luminal and non-luminal molecular subtypes of invasive breast cancer patients. To assess the clinical usefulness of radiomics signature, DCA was performed by calculating net benefits over a threshold probability range for the entire ensemble.

### 2.8. Statistical analysis

Statistical analysis was conducted using SPSS 19.0 and R software (Version 3.4.4; http://www.Rproject.org). The chi-square test was used for the analysis of categorical variables and the mean values ± standard deviations was used for the analysis of continuous variables. The “glmnet (R)” package was used to perform LASSO regression. The “Regression Modeling Strategy (RMS)” package was used to construct the radiomics signature and calibration curves. The Hosmer–Lemeshow test was performed on the“generalhoslem” package. ROC curves were plotted using the “partial Receiver Operating Characteristic (pROC)” software package. The significance level was set as two-sided *p* < 0.05.

## 3. Results

### 3.1. Demographics and MRI radiological features findings

A total of 135 patients were enrolled in our study, and demographics and MRI radiological features were collected. Table 2 summarizes the differences in demographics and MRI radiological features variables between luminal and non-luminal molecular subtypes in invasive breast cancer patients in the training and validation sets. Patients with luminal molecular subtype was no significantly different from those with non-luminal (*p* > 0.05 in the training and testing sets). After univariate and multivariate logistic regression analysis, no clinical risk factors were independent predictors of luminal and non-luminal molecular subtypes in invasive breast cancer patients (Table 3).

**Table 2:** Assessed clinical risk factors of breast cancer patients in the training and validation sets.

Characteristics	Training set (*n* = 95)		*p*-value	Testing set (*n* = 40)		*p*-value
	Luminal (*n* = 55)	Non-luminal (*n* = 40)		Luminal (*n* = 23)	Non-Luminal (*n* = 17)	
Age (y)	48.0 ± 9.7	48.6 ± 11.5	0.798	50.4 ± 11.6	50.8 ± 7.5	0.918
Breast parenchymal pattern (n)			0.226			NA
Type a	2	0		0	0	
Type b	5	7		4	4	
Type c	43	32		19	13	
Type d	5	1		0	0	
Maximum diameter (cm)	33.2 ± 16.6	33.9 ± 17.1	0.851	31.8 ± 13.1	40.9 ± 19.3	0.073
Location			0.973			1.000
Left	33	23		11	9	
Right	22	17		12	8	
DCE-TIC (n)			0.601			NA
Type I	1	0		0	0	
Type II	11	10		6	6	
Type III	43	30		17	11	
MRI-determined presence of ALN metastasis (n)			0.215			0.785
Yes	22	22		10	9	
No	33	18		13	8	

Type a: almost entirely fat; Type b: scattered fibrous glandular tissue; Type c: heterogeneous fibrous glandular tissue; Type d: extreme fibrous glandular tissue; DCE-TIC, dynamic contrast enhancement-time intensity curve; ALN, axillary lymph node.

**Table 3:** Univariate and multivariate logistic regression analysis of clinical risk factors for distinguishing luminal and non-luminal molecular subtypes of invasive breast cancer.

Variable	Univariate regression		Multivariate regression	
	OR (95%CI)	*p*-value	OR (95%CI)	*p*-value
Age	0.995 [0.957–1.035]	0.796	NA	NA
Breast parenchymal pattern	1.342 [0.976–1.032]	0.792	NA	NA
Maximum diameter	0.998 [0.934–1.022]	0.849	NA	NA
Location	0.902 [0.394–2.062]	0.807	NA	NA
DCE-TIC	1.069 [0.436–2.623]	0.885	NA	NA
MRI-determined presence of ALN metastasis	0.545 [0.239–1.243]	0.149	NA	NA

### 3.2. Feature extraction, selection, and radiomics signature building

**Figure 3:**
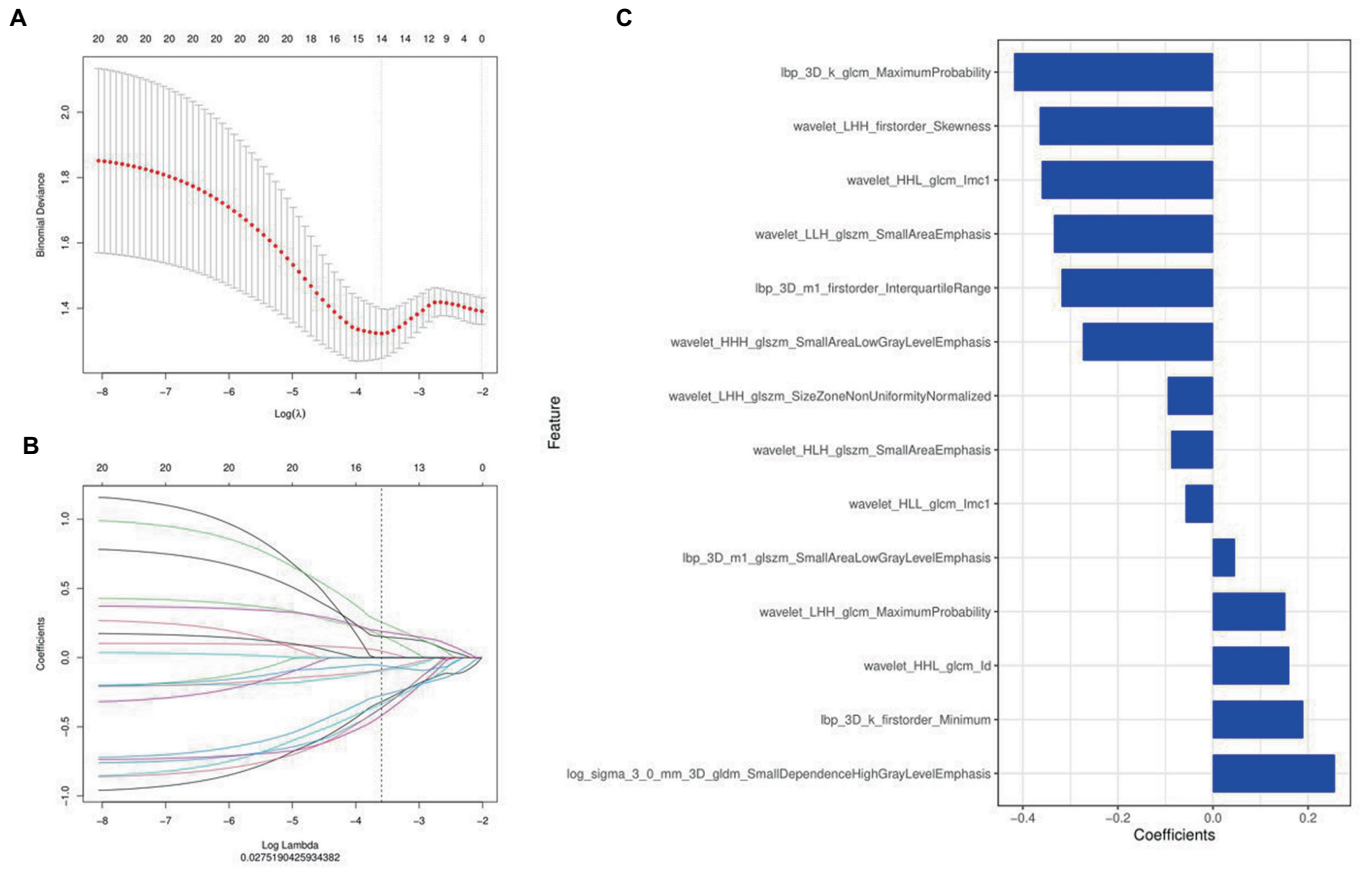
Radiomics features were selected using the least absolute shrinkage and selection operator (LASSO) regression. **(A)** Selection of tuning parameter (*λ*) in LASSO model. **(B)** LASSO coefficient profiles of radiomics features. The coefficient profiles corresponding to the selected logarithm (*λ*) values were generated by five-fold cross validation. **(C)** Selected radiomics features and their coefficients.

A total of 1,316 radiomics features were extracted from the second phase of DCE-MRI images of each invasive breast cancer patients, among which 829 features were proved to have good inter-observer and intra-observer agreement, which ICCs achieve greater than 0.75. The significant difference between luminal and non-luminal molecular subtypes in one-way ANOVA (*p* < 0.05) was enrolled into the LASSO logistic regression model to select the most valuable radiomics features (Figures 3A,B). Finally, 14 radiomics features were used to construct radiomics signature (Figure 3C).

### 3.3. Assessment of the performance of radiomics signature

**Figure 4:**
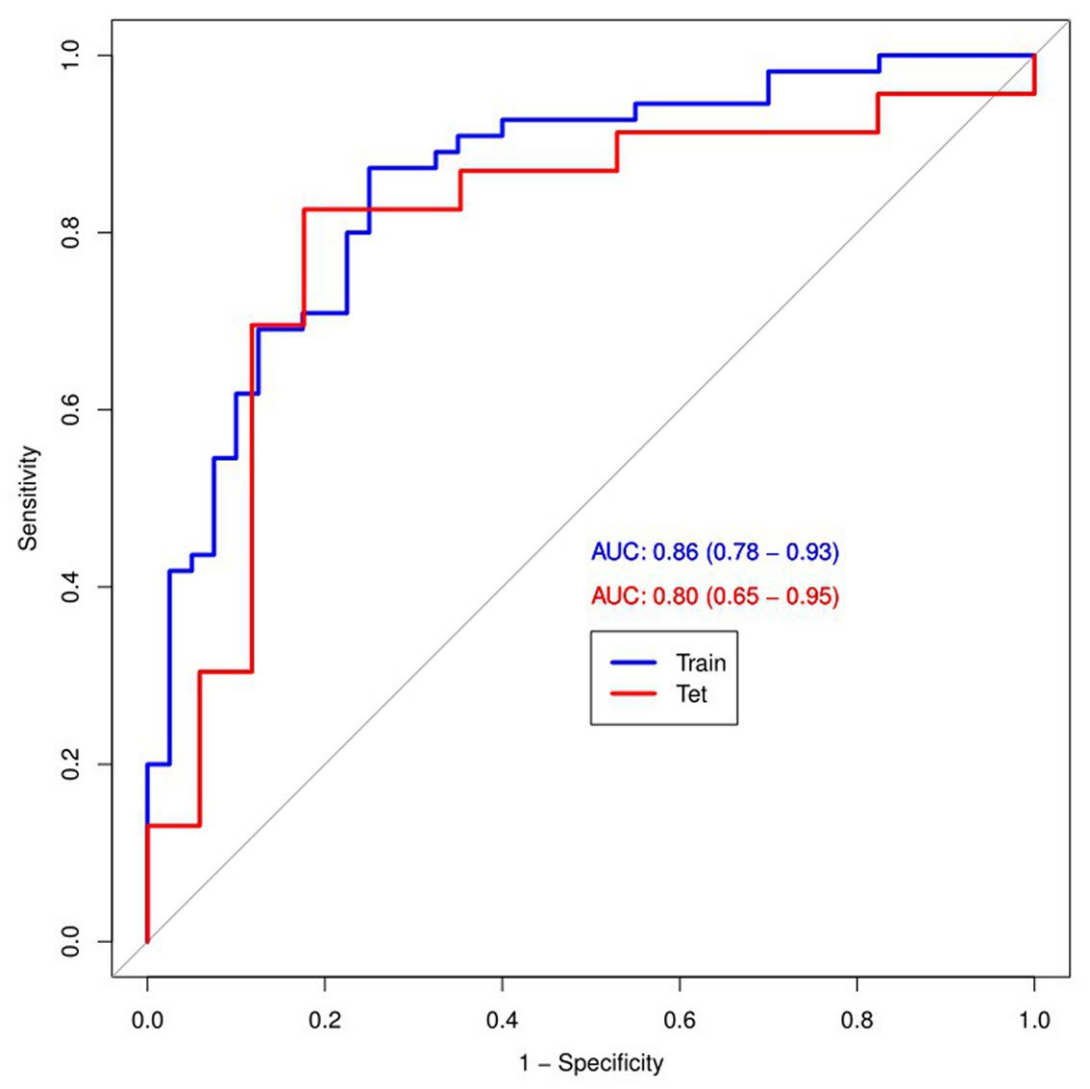
ROC curves (AUC) of the radiomics signature in the training and testing sets.

**Figure 5:**
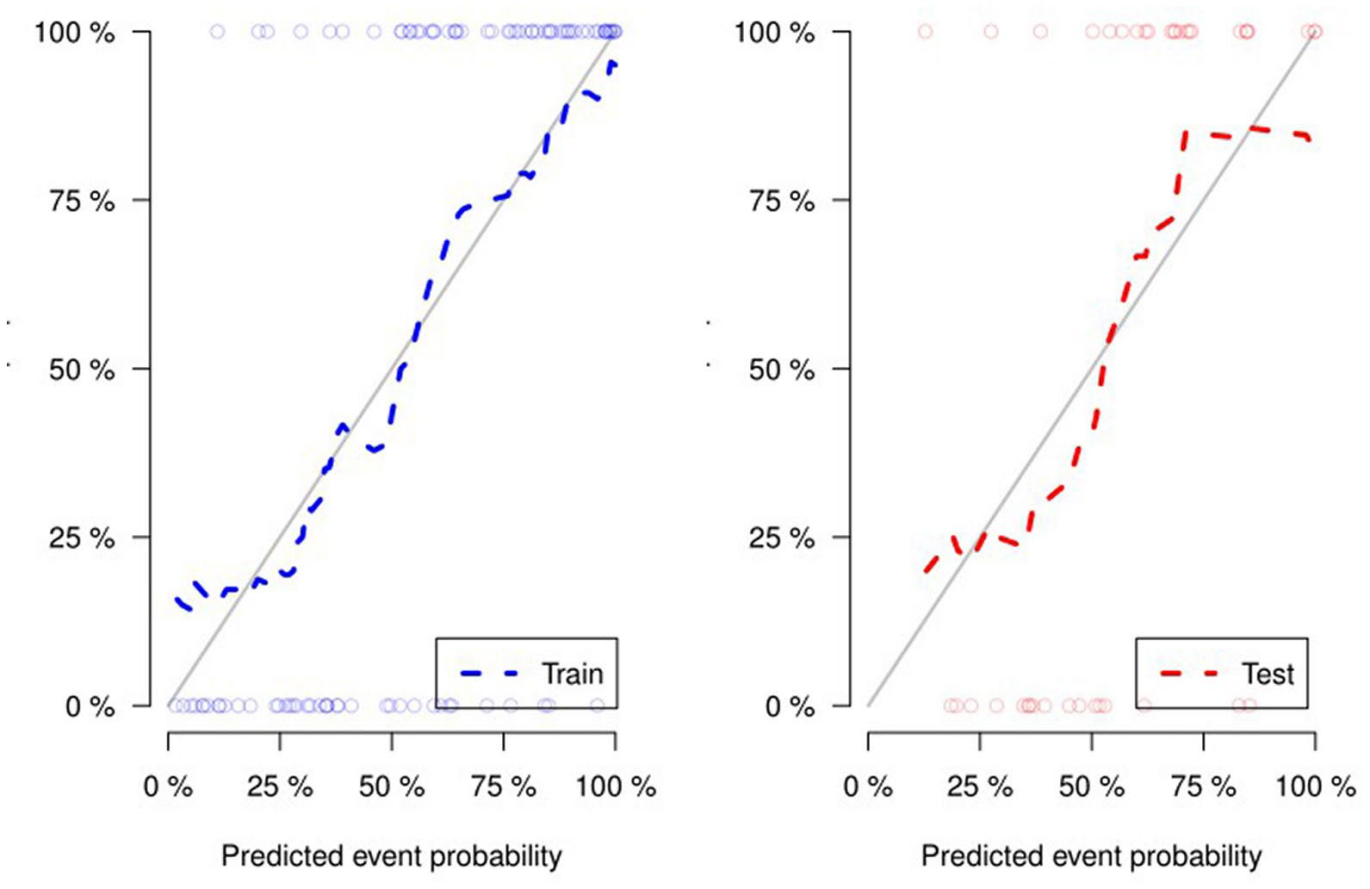
For the calibration curve of the radiomics signature, the closer the fit between the two curves, the higher the prediction accuracy.

**Figure 6:**
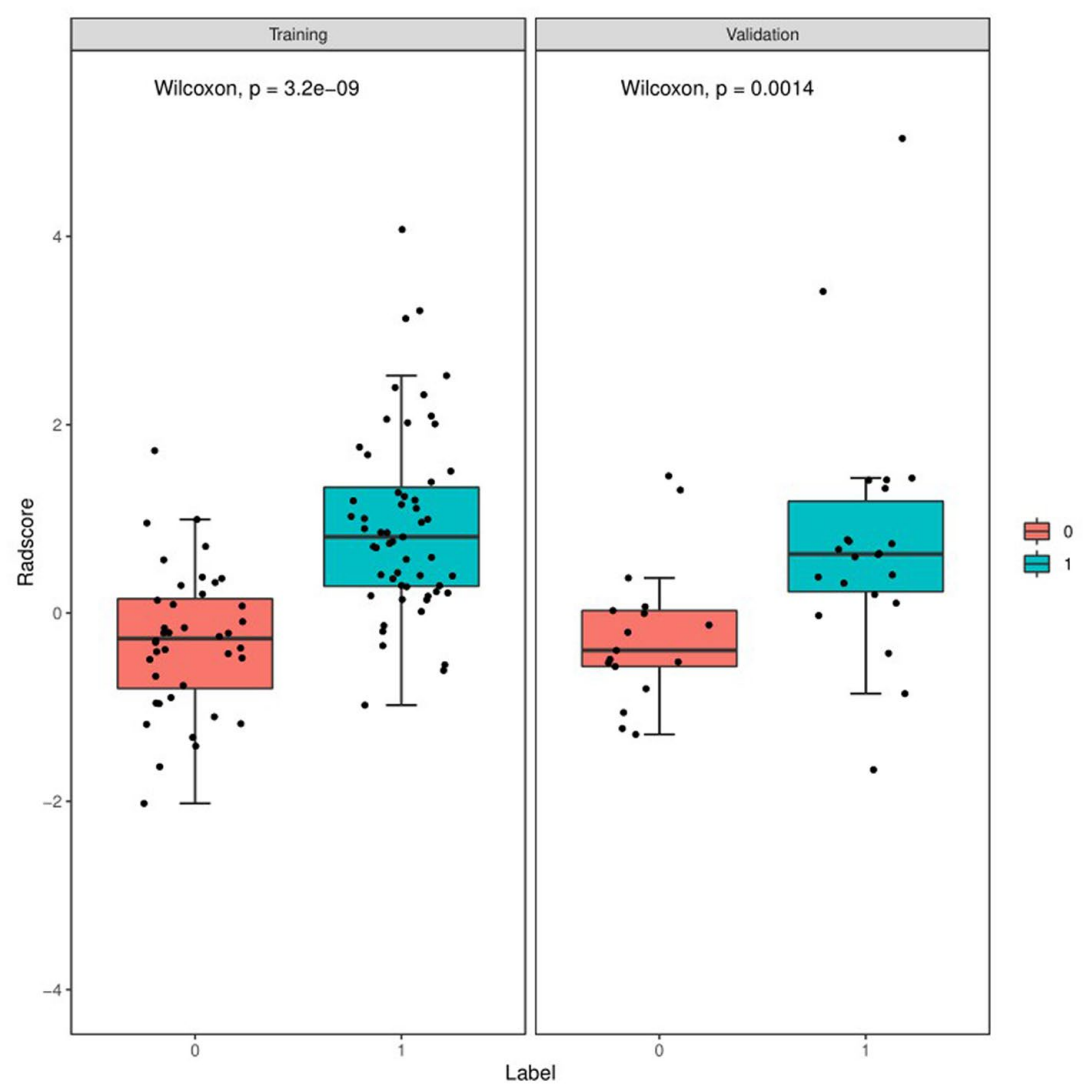
Boxplots of the radiomics score in the entire dataset shows difference between luminal and non-luminal molecular subtypes based on DCE-MRI images. The luminal group is red, the non-luminal group is blue.

Figure 4 and Table 4 present ROC curves and the radiomics signature diagnostic performance in the training and testing sets, respectively. The radiomics signature yielded an AUC value of 0.86 (95%CI 0.78–0.93) and 0.80 (95%CI 0.65–0.95) in both sets, and the accuracy, sensitivity, specificity, positive predictive value and negative predictive value were calculated. The calibration curve (Figure 5) showed a good agreement between the predicted and actual probabilities for predicting the luminal and non-luminal molecular subtypes in the training and testing sets, and the Hosmer-Lemeshow test yielded a nonsignificant statistical difference (*p* = 0.379 and 0.337). In addition, the radiomics score for each patient is shown in Figure 6. The radiomics signature were closely associated with differentiated luminal and non-luminal molecular subtypes in the training set (*p* < 0.01) and in the testing set (*p* < 0.01).

**Table 4:** Predictive performance of radiomics signature.

Model	Radiomics signature	
	Training set	Testing set
Accuracy (95%CI)	0.821 (0.729–0.892)	0.800 (0.644–0.909)
Sensitivity	0.873	0.783
Specificity	0.750	0.824
Pos. pred. value	0.828	0.857
Neg. pred. value	0.811	0.737

## 4. Discussion

In this retrospective study, we developed and validated a radiomics signature for noninvasive, individualized prediction of luminal and non-luminal molecular subtypes in invasive breast cancer patients. The radiomics signature constructed by extracting radiomics features from the second phase of DCE-MRI images showed predictive efficiency (AUC = 0.80, 95%CI =0.65–0.95) in distinguishing molecular subtypes with satisfactory reproducibility and reliability.

Accurate differentiating luminal and non-luminal molecular subtypes in patients with invasive breast cancer is an urgent need to select the most appropriate treatment. However, preoperative biopsies may mistakenly lead to errors in molecular subtypes discrimination because only small lesion areas are sampled, interobserver differences in tumor subtyping can occur even among professional breast pathologists (30, 31). Furthermore, inaccurate preoperative subtyping may lead to inadequate treatment, subsequent need for further surgery, and increased morbidity. Radiomics researches in breast imaging have focused on features extracted from DCE-MRI and their applications previously, such as the isolation of benign and malignant lesions (11), the prediction of therapeutic response (32), and the isolation of molecular subtypes (33), and results have been mixed, which may be attributed to the heterogeneity of the scanner, sequence, and features. Leithner D et al. (34) evaluated the performance of multi-parameter MRI based radiomics in conjunction with AI to evaluate breast cancer receptor status and molecular subtypes. In terms of accuracy, radiomics-based triple-negative yielded the best results with all other cancers and with luminal A and triple-negative cancers (AUC, 0.86 [0.77–0.92] and 0.80 [0.75–0.83]). However, all tumors were segmented on the largest diameter slice, and this method may not capture the heterogeneity of the tumor completely. While in the present study, we obtained better results using 3D texture features.

After multivariate logistic regression analysis, clinical risk factors we selected were not independent predictors of luminal and non-luminal molecular subtypes in invasive breast cancer patients. This result was not surprising, as it is difficult to identify by demographics and MRI radiological features alone. Son J et al. (35) aimed to predict molecular subtypes of breast cancer using radiomics signatures extracted from synthetic mammography. In multivariate analysis, radiomics signature was also the only independent predictor of the molecular subtypes. Similar founding to our study, the clinical features included age, tumor size and image features evaluated by radiologists were not independent predictors of luminal and non-luminal molecular subtypes. In addition to the conventional first-order statistical features, we employed the ITK-SNAP software to mine high-order texture parameters that are richer inside the tumors. Texture analysis appraises the relationships between pixels that generate patterns of feature organization in an image, many of which go beyond visual perception (36, 37). Five GLCM, five GLSZM and one GLDM high-order texture parameters (dependence variance) were included in the feature-screening results. The spatial structure of each tumor is different. Based on previous studies (38, 39), it’s easier to identify different molecular subtypes of breast lesions by extracting higher-order features.

In this study, the radiomics features of the second phase of DCE-MRI images of breast cancer were analyzed. Compared to mammography, DCE-MRI can provide high time, high space and high signal-to-noise ratio images for the diagnosis of breast lesions by evaluating tumor morphology and hemodynamics (40). The images in the second phase were significantly enhanced to better reflect the aggressiveness and heterogeneity of the tumor (41). It is valuable to focus on more MRI sequences in subsequent studies, and it is expected that the radiomics signature of multimodal MRI images can provide us with more useful information and further improve the predictive efficiency of the model.

The limitations of this study: (1) this is a single-center, small-sample retrospective study, and external verification of model stability and clinical applicability should be added to the multi-center data set, (2) manual lesion delineation may lead to the formation of errors, thus losing part of the image information. Therefore, more accurate lesion contour delineation methods such as semi-automatic segmentation are needed to extract the lesion characteristic values in the future, and (3) in this study, we only examined traditional radiomics analyses, and the differences in performance and robustness between our study and deep neural network-based studies in evaluating luminal and non-luminal states require further comparison.

## 5. Conclusion

In conclusion, the radiomics signature based on the second phase of three-dimensional DCE-MRI images developed in this study can be useful for differentiating luminal and non-luminal molecular subtypes. As a non-invasive, preoperative method, the radiomics signature may helpful for clinical decision-making in invasive breast cancer patients.

## Data availability statement

The raw data supporting the conclusions of this article will be made available by the authors, without undue reservation.

## Ethics statement

The studies involving human participants were reviewed and approved by Jiangxi Provincial People’s hospital’s ethics committee. Written informed consent for participation was not required for this study in accordance with the national legislation and the institutional requirements.

## Author contributions

TH and TY made the conception for this research. Data collection and analysis were performed by TH, XW, CW, YQ, and RZ. TH and HL analyzed the data and drafted the article. WD, BF, and TY reviewed/edited the manuscript. All authors contributed to the article and approved the submitted version.

## Funding

This study was supported by the National Natural Science Foundation of China (Grants No. 82160335).

## Conflict of interest

Author HL was employed by GE Healthcare.

The remaining authors declare that the research was conducted in the absence of any commercial or financial relationships that could be construed as a potential conflict of interest.

## Publisher’s note

All claims expressed in this article are solely those of the authors and do not necessarily represent those of their affiliated organizations, or those of the publisher, the editors and the reviewers. Any product that may be evaluated in this article, or claim that may be made by its manufacturer, is not guaranteed or endorsed by the publisher.
